# Evaluation of a Thermophilic, Psychrostable, and Heavy Metal-Resistant Red Sea Brine Pool Esterase

**DOI:** 10.3390/md20050274

**Published:** 2022-04-19

**Authors:** Shimaa F. Ahmed, Rehab Z. Abdallah, Rania Siam

**Affiliations:** 1 Biology Department, School of Sciences and Engineering, The American University in Cairo, New Cairo 11835, Egypt; shimaa.farag@aucegypt.edu (S.F.A.); r.abdallah@aucegypt.edu (R.Z.A.); 2 Max Planck institute for Terrestrial Microbiology, 35043 Marburg, Germany; 3 University of Medicine and Health Sciences, Basseterre, Saint Kitts and Nevis

**Keywords:** thermophilic, psychrostable, heavy metals, commercial esterases, brine pool, metagenomics

## Abstract

Lipolytic enzymes catalyze the hydrolysis and synthesis of ester compounds. They are valuable in the pulp, food, and textile industries. This study aims to comprehensively evaluate the extreme properties of a hormone-sensitive lipase (EstATII-TM) isolated from the Red Sea Atlantis II brine pool. EstATII-TM was cloned, expressed, and its biochemical activities were assessed under different conditions. EstATII-TM catalytic properties and resistance to different metal ions were compared to commercial thermophilic esterases under different temperatures. Phylogenetically, EstATII-TM was assigned to the GDSAG motif subfamily of hormone-sensitive lipase. The optimal enzyme activity was evident at a temperature of 30 °C and pH 7–8. The enzyme retained 84.9% of its activity at 0.5 M NaCl. EstATII-TM maintained 93% to 97% activity at −40 and −20 °C, respectively. EstATII-TM activity was significantly enhanced, up to 10-fold, at temperatures ranging from 45 to 65 °C in the presence of 1 mM Cu^2+^, Cd^2+^, Ba^2+^, Mn^2+^, and Zn^2+^. EstATII-TM showed superior catalytic activity and resistance-to/enhancement-by metal ions compared to two commercial thermophilic esterases. The Red Sea Atlantis II brine EstATII-TM is characterized by tolerance to high temperatures, stability to hot and cold conditions, as well as toxic heavy metal contamination, making it an ideal candidate for industrial processes.

## 1. Introduction

The Red Sea is a unique marine environment with relatively high salinity, resilient corals, and deep brine pools with unique physicochemical conditions. Out of the 25 Red Sea brine pools, the Atlantis II deep (ATII-D) diverse harsh physicochemical conditions, have attracted scientific attention for the isolation and characterization of extremophilic enzymes. ATII-D is the deepest Red Sea brine pool, characterized by a temperature of 68.2 °C, salinity of 25.7%, pH value of 5.3, and an abundance of heavy metals [1].

Extremophilic microorganisms are natural factories for extremozymes, such as ligase, esterase, nitrilase, and metal remediating enzymes [2,3,4]. These extremozymes have diverse applications in the food, pharmaceutical, and biotechnological industries [5]. Despite such potential, most commercial enzymes are mesophilic. Mesophilic enzymes lack the intrinsic properties to resist the harsh conditions associated with industrial processes and require further biological treatments associated with increased costs [6]. Hence, discovering and mining extremozymes became an area of interest in white biotechnology to reduce costs [7].

Metagenomics and functional metagenomics approaches advanced the discovery of extremophilic microorganisms and their enzymes through mining DNA, from extreme environments, for gene-encoding extremozymes with a potential role in industries [8]. Esterases and lipases (hydrolases) are heavily used in numerous industrial processes. Esterases specifically are widely used in food, beverage, perfume, pharmaceutical, textile, pulp, paper recycling, and leather industries [9]. The presence of heavy metals is a characteristic of many of these industrial processes [10]. Therefore, the need for heavy metal-stable and/or metal-dependent esterases is pressing.

Metal-dependent enzymes are abundant in nature, and they are divided into two major categories. The first category consists of enzymes with a catalysis metal-redox center. The metal ion in these enzymes directly catalyzes or assists in the catalysis reaction [11]. The second category includes enzymes that are not involved in a redox reaction. The metal ion in such enzymes either stabilizes the transition or the intermediate states of the enzyme or activates a substrate/cofactor [11]. Within the hydrolase class, the lipase and esterase enzyme families hydrolyze ester bonds; lipases hydrolyze long-chain fatty acids while esterase hydrolyzes short-chain fatty acids. As of date, only 20 hydrolases acting on ester bonds (EC 3.1.-.-) are characterized as metal-dependent enzymes in the Metal MACiE database, with only one carboxylic ester hydrolase, named phospholipase A2 [12]. Nonetheless, esterase activity’s stimulation or enhancement by heavy metals has been discussed in the literature [2,13,14,15,16,17,18,19]. Cu^2+^, Ca^2+^, Ba^2+^, Mg^2+^, Mn^2+^, and Zn^2+^ were shown to enhance the activity of 10 out of 23 esterases and hormone-sensitive lipases (HSL) by 102% to 192.9% relative activity [2,14,15,16,17,18,19]. However, this enhancement was only evident within the optimal enzyme temperature [2,14,15,16,17,18,19]. Reports on the enhancement and/or stabilization of esterase activity under unfavorably high temperatures are scarcely discussed in the literature.

In this paper, we evaluated the specific activities of a thermostable and heavy metal-resistant bacterial hormone-sensitive lipase (EstATII-TM) identified from the Atlantis II brine pool under extreme conditions, such as extreme temperatures (−80 to 65 °C), high salinity (up to 3 M NaCl), and in the presence of different metal ions [2]. EstATII-TM retained enzymatic activity under a wide temperature range; −80 °C to 45 °C. Unexpectedly, several metal ions (Cu^2+^, Ba^2+^, Cd^2+^, Mn^2+^, and Zn^2+^) enhanced EstATII-TM activity under optimal (30 °C) and at high (up to 65 °C) temperatures. We also compared EstATII-TM esterase activity with two commercially thermophilic esterases, and reported the competitive advantage of EstATII-TM in the presence of different metal ions and under various temperatures. This places EstATII-TM as an interesting enzyme in industrial processes characterized by elevated temperatures and the presence of toxic heavy metal concentrations.

## 2. Results

**Figure 1:**
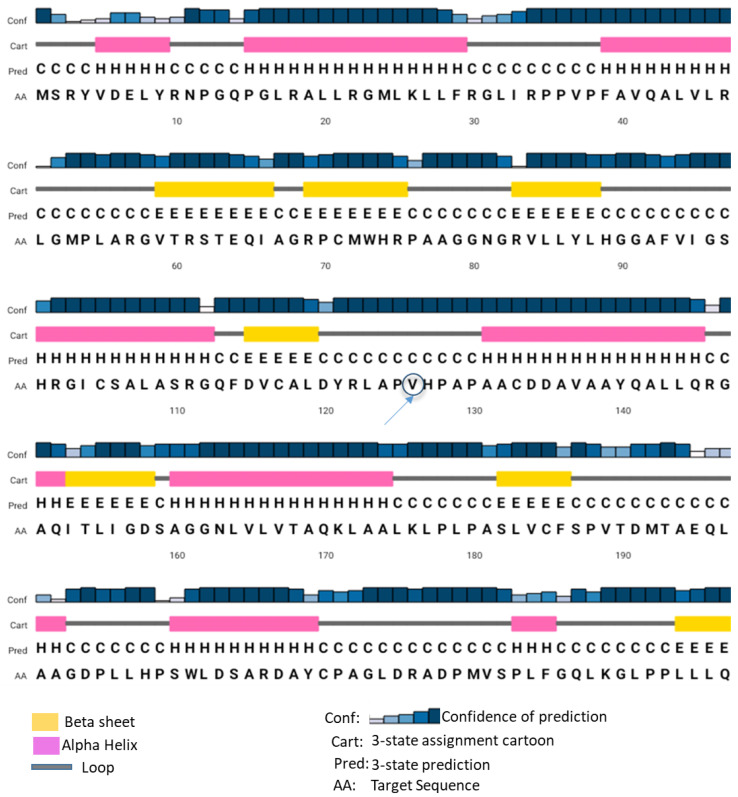
EstATII-TM secondary structure. The secondary structure of EstATII-TM is illustrated with the color codes and straight lines (beta-sheets represented in yellow, alpha-helices represented in pink, and loops represented by grey lines). Loop numbers are depicted above each loop. The mutated amino acid is identified by a circle. The secondary structure was generated by the PSIPRED protein structure prediction server.

We have previously identified and partially characterized a novel esterase (EstATII) from the Atlantis II Red Sea Brine pool by measuring the relative enzymatic activity (Mohamed et al., 2013). This study reports the biochemical activities of a missense mutant of EstATII enzyme (named EstATII-TM) by measuring specific enzymatic activities (µMole of substrate catalyzed per minute per mg of EstATII-TM). The alignment of EstATII [2] and EstATII-TM showed that there is a conservative mutation (substitution of Alanine to Valine) at amino acid position 126 (Figure S1b). This is caused by a missense mutation at nucleotide position 377 where the cytosine was substituted for thiamin (Figure S1a). The secondary structure of EstATII-TM indicates that the enzyme has 21 loops, 13 alpha-helices, and 8 beta-sheets. EstATII-TM mutation occurred in loop number 9 (Figure 1). Secondary and tertiary in-silico structure analyses and the metal ion-binding site prediction and docking server showed no difference in the structure (data not shown) or metal-binding sites of EstATII and EstATII-TM (Table S1).

### 2.1. In-Silico Characterization of EstATII-TM

**Figure 2:**
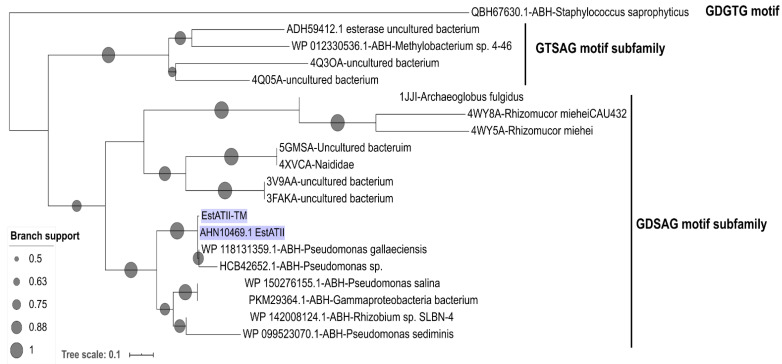
Deduced amino acid phylogenetic tree of HSL family. The dendrogram was generated by sequence alignment of the deduced amino acid sequence of EstATII-TM and EstATII (highlighted in blue) with other GTSAG motif subfamily protein sequences from the National Centre of Biotechnology (NCBI) using MUSCLE. ABH-*Staphylococcus saprophyticus* was used as an outgroup. The tree was generated using PhyML 3.1/3.0 default substitution model, and branch support values were determined by the SH-like approximate likelihood-ratio test (aLRT). Branch support values above 0.5 (50%) are shown with a grey circle. The tree was visualized and edited using the ITol web server.

The amino acid phylogenetic tree of “hormone-sensitive lipase (HSL)” placed EstATII-TM within the HSL lipolytic enzyme family and the GDSAG motif subfamily (Figure 2). All residues essential for activity were identified in EstATII-TM, including the Ser-His-Asp catalytic triad, the typical GXSXG catalytic domain, and the tetrapeptide HGGG (Figure S2). The tetrapeptide HGGG located close to the *N*-terminus is a hallmark of HSL family members with a function in stabilizing the tetrahedral intermediate.

The metal ion-binding site prediction and docking server were used to predict metal-binding sites in EstATII-TM. A total of 30 binding sites were detected for Cu^2+^, 20 for Cd^2+^, 13 for Mg^2+^, 24 for Zn^2+^, 30 for Fe^3+^, 17 for Mn^2+^, and 17 for Hg^2+^ (Table S1). The same metal ion-binding sites were predicted for EstATII (Table S1).

### 2.2. Characterization of EstATII-TM

#### 2.2.1. Cloning and Expression of EstATII-TM Gene

The EstATII-TM gene was amplified, cloned, and expressed. The purified EstATII-TM was consistent with the predicted molecular mass (55 kDa) (Figure S3).

#### 2.2.2. Qualitative Assay of EstATII-TM Enzyme Activity

EstATII-TM qualitative esterase activity was assessed by incubating the enzyme with tributyrin. The enzyme showed more efficiency toward the hydrolysis of tributyrin at 37 °C compared to 65 °C (Figure S4). Enzyme activity increased proportionally with the increase in enzyme concentration.

#### 2.2.3. Thermophilicity of EstATII-TM

**Figure 3:**
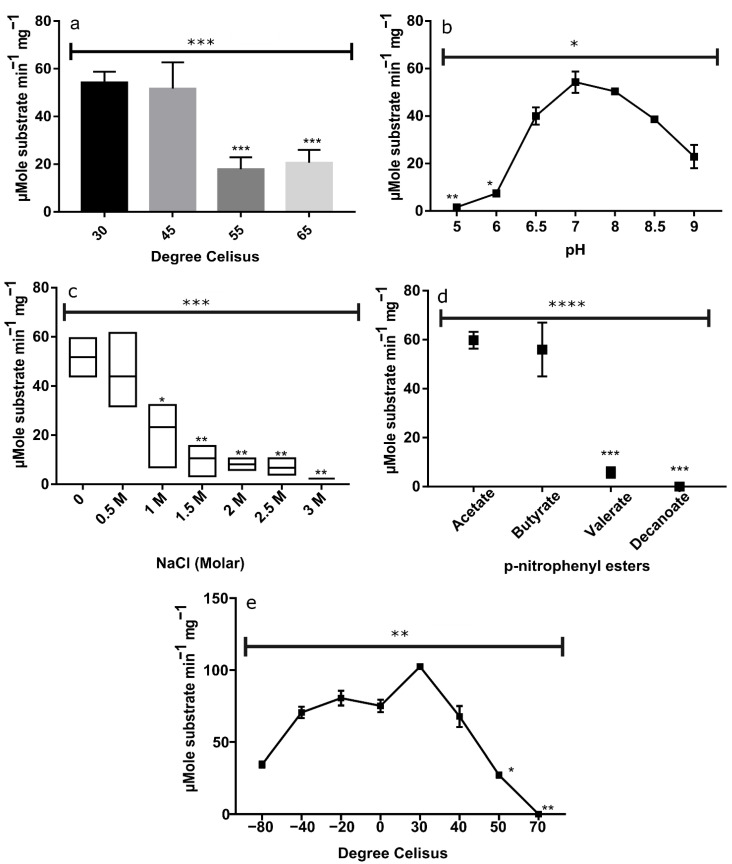
EstATII-TM characterization. (**a**) Thermophilicity, (**b**) pH tolerance, (**c**) halophilicity, (**d**) substrate specificity, and (**e**) psychro and thermostability. The graphs show the µMole of the substrate (p-nitrophenyl butyrate in (**a**–**c**,**e**) catalyzed per minute per mg of EstATII-TM. ANOVA and Kruskal–Wallis test were used to analyze the statistical difference between the means, while Dunnett and Dunn’s tests were used as a post-hoc for multiple comparisons between the different parameters and the control (control in (**a**) and (**e**) = 30 °C, (**b**) = pH 7, (**c**) = 0 M NaCl, (**d**) = butyrate). *p*-values indicate the statistical difference (*p*-value ≤ 0.05 = *, *p*-value ≤ 0.01 = **, *p*-value ≤ 0.001 = ***, *p*-value ≤ 0.0001 = ****). The ANOVA/Kruskal–Wallis *p*-values are depicted above the graphs, while Dunnett/Dunn test *p*-values are depicted above each data point.

The effect of temperature on EstATII-TM activity was determined using p-nitrophenyl butyrate as a substrate at pH 7 and a temperature range of 30–85 °C. The optimum substrate catalysis was detected at temperatures of 30 and 45 °C (Figure 3a). Interestingly at higher temperatures, the enzyme maintained 39%, and 33% of its activity at 65 and 55 °C, respectively.

#### 2.2.4. pH Effect on EstATII-TM Activity

The activity of the purified EstATII-TM was measured at various pH values (pH 3.0 to 9.5), using p-nitrophenyl butyrate at 30 °C. EstATII-TM exhibited its optimal activity at pH 7 and 8. Below pH 7, EstATII-TM activity significantly decreased by 86% (*p*-value ≤ 0.01) and 97% (*p*-value ≤ 0.05) at pH 5 and pH 6, respectively (Figure 3b).

#### 2.2.5. Halophilicity of EstATII-TM

**Figure 4:**
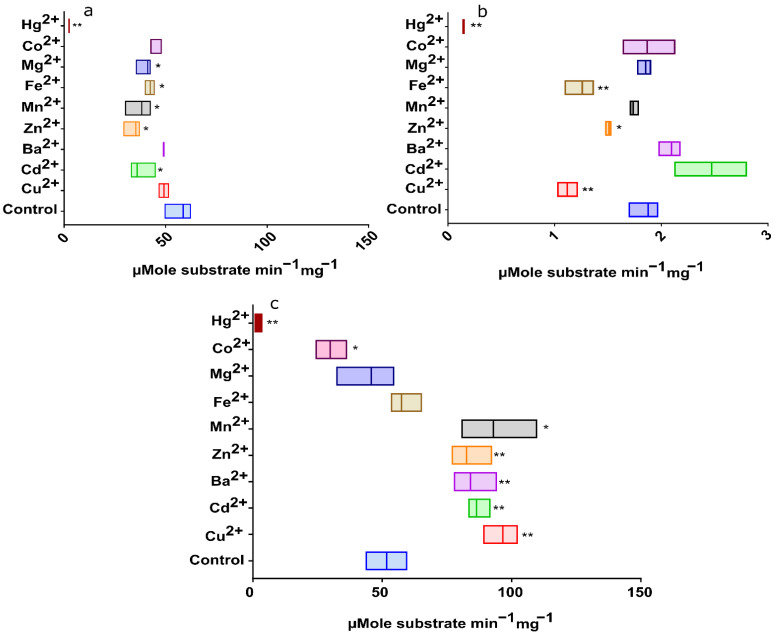
Effect of heavy metals on esterase activities under optimal conditions. (**a**) *Pseudomonas fluorescens* esterase at 25 °C and pH 7.5, (**b**) *Bacillus stearothermophilus* esterase at 65 °C and pH 7, and (**c**) EstATII-TM at 30 °C and pH 7. The graphs show the µMole of the substrate (p-nitrophenyl butyrate) catalyzed per minute per mg of esterase. An unpaired t-test with Welch’s correction was used to calculate the difference between the mean of the control reaction and the reaction with each heavy metal. *p*-values indicate the statistical difference between treatments (*p*-value ≤ 0.05 = *, *p*-value ≤ 0.01 = **. *p*-values are depicted above each data point.

Different NaCl concentrations (0.5 M to 3 M) were used to determine the salt tolerance of EstATII-TM under standard (control) assay conditions (p-nitrophenyl butyrate, pH 7 and 30 °C). At 0.5 M, the enzyme maintained 84.9% of its activity compared to the control (Figure 4c). Starting from 1 M NaCl, the enzyme activity significantly decreased with increasing NaCl concentration. The enzyme maintained ~40% (*p*-value ≤ 0.05), ~21% (*p*-value ≤ 0.01), ~16% (*p*-value ≤ 0.01), ~13% (*p*-value ≤ 0.01), and ~5% (*p*-value ≤ 0.01) activity at 1 M, 1.5 M, 2 M, 2.5 M, and 3 M, respectively, compared to the control (0 M NaCl) (Figure 3c). At 3 M NaCl, the enzyme lost ~95% of its activity (Figure 3c).

#### 2.2.6. Substrate Specificity of EstATII-TM

EstATII-TM substrate specificity was tested using p-nitrophenyl esters with varying chain lengths under standard (control) assay conditions. No significant difference was detected in the catalysis of p-nitrophenyl acetate and butyrate by EstATII-TM. Nonetheless, a sharp and significant decrease (*p*-value ≤ 0.001) in the substrate catalysis was detected with substrates containing longer acyl chains; a 90% and 100% decrease in substrate catalysis was detected with p-nitrophenyl valerate and decanoate, respectively (Figure 3d).

#### 2.2.7. Thermostability and Psychrostability of EstATII-TM

To assess the psychro and thermostability of EstATII-TM, the enzyme was incubated for 3 h at −80, −40, −20, 4, 30, 40, 50, and 70 °C, and enzyme activity was then measured under standard assay conditions. The enzyme maintained 45% of its activity following 3 h of incubation at −80 °C, and 100% activity was maintained following incubation at 4 °C for 3 h. In fact, EstATII-TM maintained 93% to 97% activity following 3 h of incubation at −40 and −20 °C, respectively (Figure 3e). On the other hand, the enzyme residual activity gradually decreased following the 3 h incubation at 40 °C and 50 °C compared to 4 °C. The enzyme lost 100% of its activity following 3 h of incubation at 70 °C (Figure 3e).

#### 2.2.8. Effect of Heavy Metals on EstATII-TM and Two Market Esterases Activities

The effect of heavy metal ions on EstATII-TM activity was assessed by incubating the enzyme at its optimal conditions with different heavy metal ions (1 mM). Similarly, the two commercial enzymes were incubated with the different heavy metal ions (1 mM) at the manufacturer’s suggested pH and temperature. Remarkably, EstATII-TM activity was significantly enhanced by the addition of Ba^2+^ (162.7%), Zn^2+^ (159.75%), Cd^2+^ (167.3044%), Cu^2+^ (186.9%), and Mn^2+^ (179.9%) when compared to the control. In contrast, both Co^2+^ and Hg^2+^ led to a significant decrease in enzyme activity with ~53% and 96%, respectively (Figure 4c). Conversely, *P. fluorescens* esterase showed a significant decrease in activity with Cd^2+^, Zn^2+^, Mn^2+^, Fe^+3^, Mg^2+^, or Hg^2+^ (Figure 4a). At the same time, *B. stearothermophilus* esterase activity decreased significantly by adding Cu^2+^, Zn^2+^, Fe^+3^, and Hg^2+^ (Figure 4b).

#### 2.2.9. Effect of Heavy Metal on EstATII-TM and Two Commercial Esterases under Thermophilic Conditions

**Figure 5:**
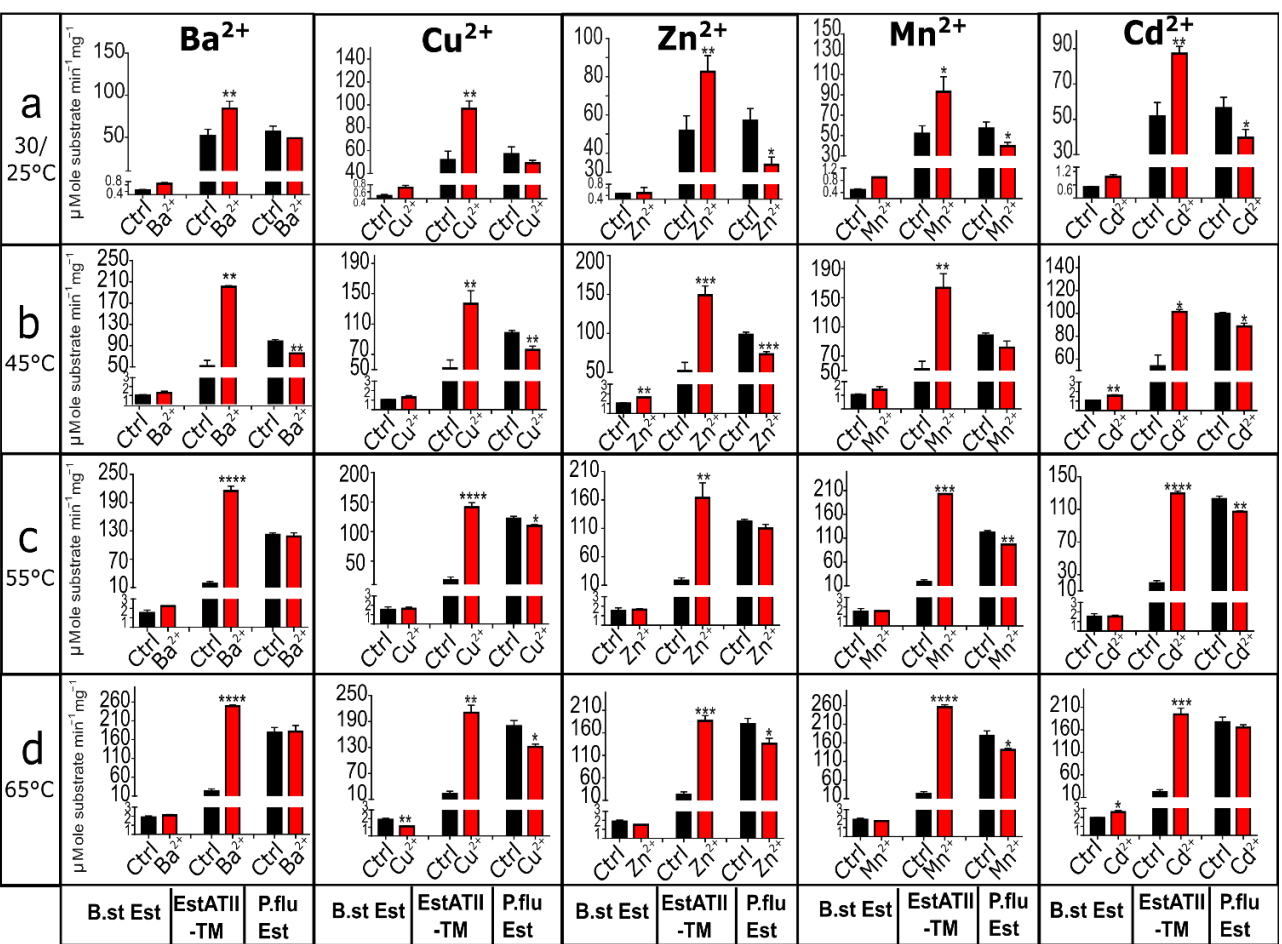
Effect of heavy metal on esterase activities under thermophilic conditions; (**a**) 30/25 °C, (**b**) 45 °C, (**c**) 55 °C, (**d**) 65 °C. The graphs show the µMole of the substrate (p-nitrophenyl butyrate) catalyzed per minute per mg of *B. stearothermophilus* esterase (B.st Est), EstATII-TM, or *P. fluorescens* esterase (P.flu Est). An unpaired t-test with Welch’s correction was used to calculate the difference between the mean of the control reaction and the reaction with each heavy metal. *p*-values indicate the statistical difference (*p*-value ≤ 0.05 = *, *p*-value ≤ 0.01 = **, *p*-value ≤ 0.001 = ***, *p*-value ≤ 0.0001 = ****). *p*-values are indicated above each data point.

Based on the significant improvement of EstATII-TM activity in the presence of Cd^2+^, Mn^2+^, Cu^2+^, Ba^2+^, and Zn^2+^, we speculated that these heavy metals might promote enzymatic activity under higher temperatures. As predicted, EstATII-TM activity increased ~10-fold at 65 °C in the presence of Mn^2+^ and Ba^2+^ compared to the control (Figure 5d). Similarly, Cd^2+^ and Zn^2+^ increased the activity by 8-fold and Cu^2+^ by 9-fold at 65 °C (Figure 5d). Likewise, when the reaction was incubated at 55 °C, a 7.5–10-fold increase in activity was detected with the addition of Cd^2+^, Mn^2+^, Cu^2+^, Ba^2+^, and Zn^2+^ (Figure 5c). At 45 °C, there was a 2–4-fold increase in activity compared to the control with Cd^2+^, Mn^2+^, Cu^2+^, Ba^2+^, and Zn^2+^ (Figure 5b).

Oppositely, the *P. fluorescens* esterase was mainly negatively affected by heavy metal ions at higher temperatures. At 45 °C, all of the tested heavy metals, except Mn^2+^, significantly decreased *P. fluorescens* esterase activity by 9.3–25% compared to the control (Figure 5b). At 55 °C, a slight but significant decrease in activity (10–20 %) was detected in the presence of Cd^2+^ (*p*-value ≤ 0.01), Mn^2+^ (*p*-value ≤ 0.01), and Cu^2+^ (*p*-value ≤ 0.05) (Figure 5c). At 65 °C, Mn^2+^ (*p*-value ≤ 0.05), Cu^2+^ (*p*-value ≤ 0.05), and Zn^2+^ (*p*-value ≤ 0.05) led to a 20–27% decrease in the activity (Figure 5d).

The esterase activity from *B. stearothermophilus* was relatively stable in the presence of the tested heavy metals at higher temperatures. Nonetheless, Cd^2+^ led to a 60 to 80% increase in activity at 45 and 65 °C, respectively (Figure 5b,d). A significant increase (83%, *p*-value ≤ 0.01) in enzyme activity was also detected when Zn^2+^ was added to the reaction at 45 °C (Figure 5b). Oppositely, Cu^2+^ led to a significant decrease (~50%, *p*-value ≤ 0.01) in activity at 65 °C (Figure 5d).

### 2.3. Enzyme Kinetics

**Figure 6:**
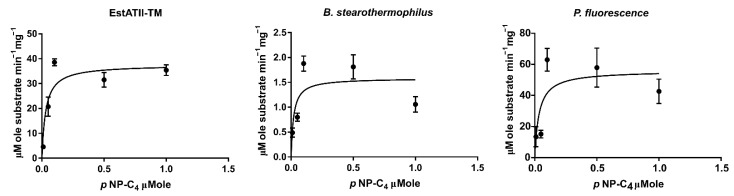
Nonlinear regression applied to Michaelis–Menten kinetics for EstATII-TM, *P. fluorescens*, and *B. stearothermophilus* esterases. The graphs represent the enzyme activity = µMole of the substrate (p-nitrophenyl butyrate) catalyzed per minute per mg of enzymes.

The Michaelis–Menten enzyme kinetics of EstATII-TM and the two commercial esterases were attained by measuring the rate of p-nitrophenyl butyrate hydrolysis at various substrate concentrations (0.01, 0.05, 0.1, 0.5, and 1 mM) and optimal reaction conditions. The *K_M_* was 0.03032, 0.01983, and 0.03504 µM while Vmax was 37.55, 59.81, and 1.584 units/g for EstATII-TM, esterase from *B. stearothermophilus*, and esterase from *P. fluorescens*, respectively (Figure 6 and Table 1). EstATII-TM had the highest catalytic rate (*Kcat*) and efficiency (*Kcat*/*K_M_*) (Table 1).

## 3. Discussion

Extreme environments represent a valuable source of extremozymes that can be utilized in diverse industries. Here, we present a comprehensive evaluation of the biochemical characteristics of a hormone-sensitive lipase EstATII-TM, from the Red Sea Atlantis II brine pool. Atlantis II brine pool is the deepest Red Sea brine pool, at a depth of 2100 m below the surface of the sea, characterized by a high temperature that could reach 68.2 °C, pH of 5.3, the salinity of up to 25.7%, and an abundance of heavy metals [1].

Our results show that EstATII-TM belonged to the carboxylesterases H family (HSL) with a conserved GDSAG motif (Figure 2 and Figure S2). The enzyme was closely related to *Pseudomonas* sp. Esterases. EstATII-TM preferentially hydrolyzed short-chain esters (C2 and C4); nonetheless, it metabolized tributyrin (C15) (Figure 3 and Figure S4) when its concentration increased 10-fold (up to 0.252 µM) (Figure S4). Bacterial hormone-sensitive lipases have wide substrate preferences; some have a preference for C2:C6, such as HSL from *Salinisphaera* sp. [20] P7-4 and metagenomic studies uncultured bacterium [21,22], while others have an optimal activity with C8:C12 esters [23,24]. Even though EstATII-TM activity showed higher catalytic activity with short-chain esters, it showed the highest catalytic efficiency and rate in comparison to the two commercial enzymes tested when using the long-chain ester p-nitrophenyl butyrate (p NP-C4) as a substrate. Overall, EstATII-TM catalytic efficiency and *Kcat* are among the highest in its family [20,23,25,26,27]. EstATII-TM optimal pH was 7, nonetheless, it maintained >90% and >40% of its activity at pH 8 and pH 9, respectively. The alkali-stability property of EstATII-TM makes it a good potential candidate for several industrial processes. A moderate alkalophilic condition was detected in other bacterial HSL isolated from *Glaciozyma antarctica* [24], *Archaeoglobus fulgidus* [27], *Alicyclobacillus acidocaldarius* [27], *Staphylococcus xylosus* [28], *Salimicrobium* sp. LY19 [29], and metagenomic DNA [21,22,30]. EstATII-TM retained the majority of its activity (84.6%) under 2.9% NaCl (0.5 M), and it maintained 45% of its activity at 5.8% NaCl (1 M); this suggests that it is a moderately halotolerant enzyme. However, EstATII-TM is not halophilic since we witnessed the decrease in EstATII-TM enzyme activity in the presence of higher salt concentration, and loss of activity at 3 M NaCl, which has likely disrupted the enzyme 3D structure confirmation [31].

Our enzyme could be characterized as mesophilic to slightly thermophilic with optimum temperatures ranging from 30 to 45 °C. Nonetheless, the enzyme remained active at 65 °C. The decrease in the enzyme thermophilicity reported in this study, from its wild type version EstATII is likely due to the different techniques used in the two studies; specific activity (this study) vs. relative activity [2]. Previously [2], the relative enzyme activity was represented by the ratio of the enzyme activity and the activity of control. In this study, we present the enzyme activity in µMole of substrate catalyzed per minute per mg of EstATII-TM. On the other hand, the enzyme was stable for up to 3 h at a wide range of temperatures (from −20 to 4 °C). To our knowledge, EstATII-TM is the first HSL that can withstand below freezing temperatures, including HSL isolated from psychrophilic microbes, such as *Salinisphaera* sp. P7-4 [20], *Salimicrobium* sp. LY19 [29], and *Psychrobacter* sp. TA14 [32], or cold environments [30]. The stability of EstATII-TM under cold and room temperature provides it with a storage advantage that could be useful in industrial settings.

A core and unique characteristic of EstATII-TM is its resistance and enhancement by a variety of heavy metals ions, such as copper, cadmium, barium, zinc, manganese, iron, and magnesium [2]. Several bacterial HSL esterases showed resistance to cadmium, manganese, iron, and magnesium [24,25,26,29]. EDTA did not affect EstATII relative activity, suggesting that EstATII-TM is not a metalloenzyme [2]. Nonetheless, Cu^2+^, Cd^2+^, Mg^2+^, Zn^2+^, Fe^+3^, Mn^2+^, and Hg^2+^ binding sites were detected in the EstATII-TM (Table S1). The activating effects of Cu^2+^, Cd^2+^, Ba^2+^, Zn^2+^, and Mn^2+^, and the presence of their binding sites indicate that EstATII-TM is a metal-activated but not metal-dependent enzyme. These metals are likely to play a role in the 3D structure stability of EstATII-TM [26,32]. A similar effect was reported in a recent study of an HSL isolated from *Staphylococcus saprophyticus* AG1 [26]. It is important to note that even though the activation of HSL and esterases by metal ions was reported before, activation by Cu^2+^ and Zn^2+^ is a unique trait for EstATII-TM; these metal ions are usually inhibitory for other HSL [23,24,25,26,29]. Further studies are needed to understand the heavy metal resilience properties of EstATII-TM compared to other HSLs. EstATII-TM was superior to the two commercial enzymes used in this study with regard to heavy metal resistance. The inhibition of EstATII-TM activity by mercury and cobalt ions is likely due to mercury binding to the thiol group and cobalt binding to the sulfhydryl group in cysteine residues [33,34].

Even though several esterases and HSL are activated to some degree by metal ions, these activations were constantly reported at the enzyme’s optimal temperature. The positive effect of metal ions on the stability of esterases and HSL under unfavorable temperatures is seldom described in the literature. EstATII-TM demonstrated a remarkable enhancement in enzymatic activity, from a 2 to 10-fold increase, under thermophilic conditions (45 to 65 °C) in the presence of Cu^2+^, Cd^2+^, Ba^2+^, Zn^2+^, or Mn^2+^. This immense increase in activity in the presence of metal ions under thermophilic conditions is unique to EstATII-TM within the HSL enzymes previously described in the literature [22,25,26,27,30,32,35,36]. The increase in EstATII-TM thermophilicity in the presence of Cu^2+^, Cd^2+^, Ba^2+^, Zn^2+^, and Mn^2+^ could infer that these metal ions play a role in the enzyme structural stabilization. This hypothesis is supported by the structural stability reported with lipases in the presence of Ca^2+^ and Zn^2+^ under elevated temperatures [28,37,38]. Nonetheless, further research should be performed to understand the role of heavy metals in the structural thermostability of EstATII-TM under high temperatures. In comparison to the two commercial esterases, the enhancing effect of metal ions on the enzyme’s activity under thermophilic conditions was mainly seen in EstATII-TM, with the exception of the slight, yet significant, increase in the activity of the *B. stearothermophilus* esterase at 65 °C in the presence of Cd^2+^.

## 4. Materials and Methods

### 4.1. Environmental DNA Isolation and Fosmid Library Construction

Environmental DNA was extracted from 0.1 μm filters of the low convective layer of the Atlantis II brine pool and the Copy Control Fosmid Library Production Kit (Epicentre) was used to construct the fosmid library. Tributyrin agar plated were used to screen fosmid clones. One positive clone was subjected to fosmid DNA extraction and pyrosequencing, and the EstATII gene was identified. EstATII gene sequence was used to design primers to amplify EstATII from the fosmid clone. Amplified EstATII was cloned into a p-GEM vector. This was carried out in 2013 by Mohamed Y and coauthors [2].

### 4.2. Cloning of the ESTAII-TM Gene

The EstATII-TM was amplified from a p-GEM cloning vector containing the EstATII ORF [2] using the forward prime (EstF) 5′-ATG TCC AGG TAC GTT GAT GAG C-3′, reverse primer (EstR) 5′-TCA GCT TAC CGA GTC GGT CT-3′, and DreamTaq Master Mix (Thermo Fisher SCIENTIFIC, Waltham, MA, USA) according to the manufacturer’s instructions. A 945 bp band was observed on an agarose gel (Figure 3a). The amplified ORF was cloned into the pET-SUMO vector (Thermo Fisher SCIENTIFIC, USA). The recombined expression plasmid was transformed into the TOP10 Chemically Competent *E. coli* cells, as previously stated [39]. Both PCR (using ORF’s forward primer and the vector’s reverse primer) and double restriction digestion with spaI and HindIII were performed to verify the correct orientation of the EstATII-TM ORF. The plasmid with the EstATII-TM gene inserted in the correct frame (colony C4) was used for expression in *E. coli* BL-21 (DE3) (Figure 3b).

### 4.3. Gene Sequences and Computational Analysis

According to the manufacturer’s instructions, both p-GEM and pET-SUMO plasmids were extracted by QIAprep Spin Miniprep Kit (QIAGEN, Germantown, MD, USA). Both plasmids were sequenced by Sanger sequencing using the plasmids’ forward and reverse primers (Macrogen, Seoul, Korea). A missense mutation was detected in the ORF present in both plasmids; the mutated enzyme was referred to as EstATII-TM. This unintentional mutation likely occurred by random mutagenesis for >7 years of storage at −80 freezer.

EstATII [2] and EstATII-TM nucleotide and amino acid sequence alignment was performed using EMBOSS Stretcher (http://emboss.open-bio.org/) and visualized by Boxshade, while Clustal W was used for multiple sequence alignment generation [40,41]. The phylogenetic tree was generated based on a MUSCLE alignment of the deduced amino acid sequence from EstATII-TM and EstATII and other protein sequences from the National Centre of Biotechnology (NCBI) [42]. The tree was generated by PhyML 3.1/3.0 using the default substitution model [43]. The branch support values were based on the SH-like approximate likelihood-ratio test (aLRT) [43]. The tree was visualized and edited using the ITol web server [44].

The secondary structure was generated using PSIPRED Protein Structure Prediction Server [45]. Metal-binding sites in EstATII-TM were predicted by the MIB: metal ion-binding site prediction and docking server [46].

### 4.4. Expression and Purification of His Tagged EstATII-TM Esterase

The recombinant pET-SUMO was transformed into chemically competent *E. coli* BL21 cells grown on LB agar plates supplemented with 50 μg/mL kanamycin (Sigma Aldrich, St. Louis, MI, USA). The pre-culture used for recombinant protein expression was carried in a 10 mL LB media with kanamycin. Cultures were incubated overnight at 37 °C with orbital shaking at 200 rpm. The pre-culture was used to inoculate 1l of kanamycin supplemented LB media. The culture was grown until an OD600 of 1 was reached. Recombinant protein expression was induced by adding 0.5 mM isopropyl-β-D-1-thiogalactopyranoside (IPTG) (Sigma Aldrich, St. Louis, MI, USA) and incubation at 37 °C for 3 h. The cell pellet was collected by centrifugation at 10,000 rpm for 10 min at 4 °C. The pellet was then resuspended in a 7 mL lysis buffer (20 mM sodium phosphate, 0.5 M NaCl, 20 mM imidazole, pH 7.4) and disrupted using sonication for 15 cycles (30 s each cycle). Cell debris was centrifuged at 9000 rpm for 1 h at 4 °C. There was a negligible EstATII-TM protein in the insoluble fraction (data not shown). The supernatant was loaded onto a His Trap HP His tag protein purification column (Amershams Biosciences, UK) previously charged with NiCl2 and equilibrated according to the manufacturer’s instructions. Finally, the protein was eluted in four fractions using an elution buffer (20 mM sodium phosphate, 0.5 M NaCl, 500 mM imidazole, pH 7.4). Enzyme fractions were analyzed by SDS-PAGE. Fractions containing the protein of interest were dialyzed against sodium phosphate buffer pH 7.4 to remove the imidazole. Purified EstATII-TM protein concentration was measured by the Pierce™ BCA Protein Assay Kit (Thermo Fisher Scientific, USA) according to the manufacturer’s instructions. The purified protein fractions were stored at 4 °C.

### 4.5. Enzyme Characterization

#### 4.5.1. Qualitative Assay

The tributyrin cup-plate method was used to assess EstATII-TM activity qualitatively [47]. Two tributyrin plates were inoculated with the purified EstAII-TM at different concentrations (0.042, 0.0595, 0.084, and 0.252 µM), and phosphate buffer was used as a negative control. Plates were incubated at 65 °C and 37 °C. A clear zone indicated the presence of esterase activity.

#### 4.5.2. Quantitative Assay

EstATII-TM activity was determined by measuring the p-nitrophenol released from esterase-catalyzed hydrolysis as previously described [48]. The production of p-nitrophenol was continuously monitored at 410 nm by Shimadzu 1800 UV-spectrophotometer (Shimadzu Scientific Instruments (SSI), Columbia, MD, USA) with a thermal controller. Esterase activity was measured by a standard assay at 30 °C, with 0.1 mM p-nitrophenyl butyrate (Sigma Aldrich, St. Louis, MI, USA) as a substrate in 50 mM phosphate buffer pH 7; then the reaction was started by adding 0.025 µM of the purified enzyme [2]. The activity was indicated in µM of substrate converted per mg of protein per minute (IU). Blank reactions were performed with every measurement to subtract non-enzymatic hydrolysis of substrates from the results. All the experiments were performed in triplicate (except pH 6.5, 8, and 8.5 were made in duplicate).

#### 4.5.3. Effect of Different Temperatures on Enzyme Activity and Thermostability

The effect of different temperatures (thermophilicity) on EstATII-TM activity was determined by performing the hydrolytic reaction at different temperatures ranging from 30 to 65 °C. The reaction mixture was continuously monitored for the production of p-nitrophenol.

The thermostability and psychrostability assays were conducted by incubating EstATII-TM for 3 h at each of the following temperatures: −80 °C, −40 °C, −20 °C, 4 °C, 30 °C, 40 °C, 50 °C, and 70 °C. Following the incubation period, enzyme activity was determined, as previously mentioned.

#### 4.5.4. Effect of Different pH on Enzyme Activity

The effect of pH on EstATII-TM was determined by performing the hydrolytic reaction at 30 °C and different pH concentrations. A total of 50 mM of sodium acetate buffer was used for pH range 3 to 5.5; 50 mM of sodium phosphate buffer was used in the reaction mixtures for pH range 6 to 7.5; 50 mM Tris-HCL buffer was used in the reaction mixtures with a pH range of 7.5 to 12.

#### 4.5.5. Effect of Halophilicity on Enzyme Activity

The activity of EstATII-TM under halophilic conditions was determined by adding NaCl to the enzyme mixture at different concentrations (0–3 M) under standard assay conditions (30 °C and pH 7). The enzyme activity was measured as previously mentioned.

#### 4.5.6. Enzyme Substrate Specificity

The substrate specificity of EstATII-TM was determined by performing the hydrolytic reaction under standard assay conditions using different p-nitrophenyl esters with varying fatty acid chain lengths. The short-chain fatty acid esters were PNP-acetate (C2), PNP-butyrate (C4), and PNP-valerate (C5), and the long-chain fatty acid ester was PNP- decanoate (C10).

#### 4.5.7. Effect of Metal Ions

Cations (Cd^2+^, Mg^2+^, Cu^2+^, Zn^2+^, Co^2+^, Mn^2+^, Hg^2+^, Fe^+3^, or Ba^2+^) were added to the reaction mixture at a final concentration of 1 mM to assess the effect of each metal ion on EstATII-TM activity. The enzyme activity was measured under standard assay conditions (30 °C and pH 7) using PNP-butyrate as substrate. Cations with EstATII-TM enhancing effects (Cd^2+^, Cu^2+^, Zn^2+^, Mn^2+^, or Ba^2+^) were incubated with the reaction mixture at 45 °C, 55 °C, and 65 °C. Enzyme activity was measured as previously mentioned.

#### 4.5.8. Effect of Metal Ions on Two Commercial Esterases

The previously mentioned heavy metals were added in 1 mM (final concentration) to a reaction mixture containing either 0.025 µM of *Pseudomonas fluorescens* esterase (Sigma Aldrich, St. Louis, MI, USA, or 0.625 µM of *Bacillus stearothermophilus* esterase (Sigma Aldrich, St. Louis, MI, USA). The hydrolytic reaction was incubated at 25 °C/pH 7.5 for *P. fluorescens* esterase and 65 °C/pH 7 for *B. stearothermophilus* esterase, according to the manufacturer’s instructions. The substrate in the reaction in the two reaction mixtures was PNP-butyrate. Cations with EstATII-TM enhancing effect (Cd^2+^, Cu^2+^, Zn^2+^, Mn^2+^, or Ba^2+^) were also incubated with the two esterases at 45 °C, 55 °C, 65 °C.

#### 4.5.9. Enzyme Kinetics

Reaction rates were investigated at varying p-nitrophenyl butyrate concentrations (0.01–1 mM) at the enzymes optimum physical conditions; 30 °C and pH 7.5 for EstATII-TM, 70 °C and pH 7.5 for the esterase from *P. fluorescens* (Sigma Aldrich, St. Louis, MI, USA) [19], and 65 °C and pH 7 esterase from *B. stearothermophilus* [49]. The Michaelis–Menten constant (*K_M_*) and the maximum reaction velocity (Vmax) and *Kcat* were estimated using the non-linear regression equations in Graphpad prism 7.

#### 4.5.10. Statistical Analysis

The variation in means between treatments in the characterization experiments was calculated using the ANOVA test when the data were normally distributed or the Kruskal–Wallis test when the data were not normally distributed [50,51]. Dunnett and Dunn’s tests were used as a post-hoc for multiple comparisons between the different parameters in each experiment [52,53]. The difference between the control reaction and heavy metal supplemented reactions were calculated using an unpaired t-test with Welch’s correction [54]. All statistical analyses were performed using Graphpad prism 7.

#### 4.5.11. Data Deposition

The nucleotide accession number for EstATII (wild type) is KC958722.

## 5. Conclusions

In summary, here we evaluated the extreme biochemical properties of a hormone sensitive lipase from the Atlantis II Red Sea brine pool (EstATII-TM). Using different analytical tools, we show additional extreme properties of the Atlantis II Red Sea brine pool that were not reported in the wild type protein form (EstATII) [2]. EstATII-TM shows stability under cold temperature, alkali-stability, halotolerant, and a strong enhancement of its esterase activity in the presence of specific metal ions, even under thermophilic conditions. EstATII-TM was superior in its catalytic activity and resistance to heavy metals in comparison to two commercially thermophilic esterases. The results presented here show that EstATII-TM is a strong esterase candidate for commercial use. Additionally, it could be considered an ideal esterase for industrial processes involving high temperatures and contamination by heavy metal ions.

## Figures and Tables

**Table 1:** Nonlinear regression applied to Michaelis–Menten kinetics for EstATII-TM, *P. fluorescens*, and *B. stearothermophilus* esterases under optimum conditions.

Enzyme	V_max_ (μmol∙min^−1^∙mg^−1^)	*K_M_* (μmol)	*K_cat_* (s^−1^)	*K_cat_/K_M_* (μmol^−1^ s^−1^)
EstATII-TM	37.55 (±2.84)	0.03 (±0.01)	4.65 × 10^4^ (±9.27 × 10^3^)	1.5 × 10^6^
*B. stearothermophilus*	1.58 (±0.19)	0.02(±0.01)	2.54 (±0.30)	0.01
*P. fluorescens*	55.93 (±7.83)	0.04 (±0.02)	2.7 × 10^4^ (±3.3 × 10^4^)	7.70 × 10^5^

± represents standard of error.

## Data Availability

The data presented in this study are available in Figure 1, Figure 2, Figure 3, Figure 4, Figure 5, Figure 6 and Figures S1–S4 and Table S1.

## Supplementary Materials

The following supporting information can be downloaded at: https://www.mdpi.com/article/10.3390/md20050274/s1, Figure S1: Est-ATII and Est-ATII-TM DNA and protein alignments, Figure S2: Amino acids sequence alignments of EstATII, EstATII-TM, and enzymes from the HSL family, Figure S3: EstATII-TM cloning and expression, Figure S4: The qualitative assay of EstATII-TM using the cup-plate method and tributyrin (triglyceride) as a substrate, Table S1: Metal ions binding sites based on the Metal Ion-Binding site prediction and docking server (MIB).
