# Supplementary material for: Evaluation of a Thermophilic, Psychrostable, and Heavy Metal-Resistant Red Sea Brine Pool Esterase
Source: Mar Drugs. 2022 Apr 19;20(5):274. doi: 10.3390/md20050274 (PMC9144027; doi:10.3390/md20050274)
Supplement: Supplementary file 1 [file marinedrugs-20-00274-s001.zip › marinedrugs-1649389-supplementary.pdf]

Journal: Marine Drugs

Title: Evaluation of a thermophilic, psychrostable and heavy metal resistant Red Sea brine pool esterase

Shimaa F. Ahmed[1†], Rehab Z. Abdallah[1,2†], Rania Siam[1,3,*]

[1] Biology Department, School of Sciences and Engineering, The American University in Cairo, New Cairo 11835, Egypt; shimaa.farag@aucegypt.edu (S.F.A.); r.abdallah@aucegypt.edu (R.Z.A.)
[2] Max Planck institute for Terrestrial Microbiology, 35043 Marburg, Germany
[3] University of Medicine and Health Sciences, Basseterre, Saint Kitts and Nevis
***** Correspondence: rsiam@aucegypt.edu
†These authors contributed equally to this work.

(a)

```
ESTAII-TM    ATGTCCAGGTACGTTGATGAGCTGTATCGCAACCCGGGCCAACCCGGCCTGCGCGCCCTG
ESTAII       -TGTCCAGGTACGTTGATGAGCTGTATCGCAACCCGGGCCAACCCGGCCTGCGCGCCCTG
              ***********************************************************

ESTAII-TM    CTGCGCGGCATGCTGAAACTGCTGTTCCGGGGTTTGATCCGTCCGCCCGTGCCCTTTGCC
ESTAII       CTGCGCGGCATGCTGAAACTGCTGTTCCGGGGTTTGATCCGTCCGCCCGTGCCCTTTGCC
             ************************************************************

ESTAII-TM    GTGCAGGCACTGGTGCTGCGCCTGCTGACCCTCGGCATGCCGCTGGCCAGAGGCGTGACC
ESTAII       GTGCAGGCACTGGTGCTGCGCCTGCTGACCCTCGGCATGCCGCTGGCCAGAGGCGTGACC
             ************************************************************

ESTAII-TM    CGCAGCACCGAGCAGATCGCCGGACGGCCCTGTATGTGGCACCGCCCGGCCGCTGGCGGC
ESTAII       CGCAGCACCGAGCAGATCGCCGGACGGCCCTGTATGTGGCACCGCCCGGCCGCTGGCGGC
             ************************************************************

ESTAII-TM    AACGGCCGCGTGCTGCTGTACCTGCATGGCGGCGCCTTCGTCATCGGCTCCCCGCAGACC
ESTAII       AACGGCCGCGTGCTGCTGTACCTGCATGGCGGCGCCTTCGTCATCGGCTCCCCGCAGACC
             ************************************************************

ESTAII-TM    CACCGCGGCATCTGCTCGGCGCTCGCCAGCCGTGGTCAGTTTGATGTCTGCGCACTCGAT
ESTAII       CACCGCGGCATCTGCTCGGCGCTCGCCAGCCGTGGTCAGTTTGATGTCTGCGCACTCGAT
             ************************************************************

ESTAII-TM    TACCGACTGGCGCCGGTGCACCCGGCACCGGCGGCCTGTGACGATGCGGTCGCCGCCTAT
ESTAII       TACCGACTGGCGCCGGCGCACCCGGCACCGGCGGCCTGTGACGATGCGGTCGCCGCCTAT
             **************** *******************************************

ESTAII-TM    CAGGCCCTGCTGCAGCGAGGCTATGCGCCCGCGCAGATCACCCTGATCGGCGATTCGGCG
ESTAII       CAGGCCCTGCTGCAGCGAGGCTATGCGCCCGCGCAGATCACCCTGATCGGCGATTCGGCG
             ************************************************************

ESTAII-TM    GGCGGCAACCTGGTACTGGTGACCGCGCAGAAACTGGCCGCGCTCAAGCTGCCGCTGCCG
ESTAII       GGCGGCAACCTGGTACTGGTGACCGCGCAGAAACTGGCCGCGCTCAAGCTGCCGCTGCCG
             ************************************************************

ESTAII-TM    GCCTCGCTGGTCTGCTTTTCACCGGTCACCGACATGACCGCCGAACAGCTGCACGCGCCT
ESTAII       GCCTCGCTGGTCTGCTTTTCACCGGTCACCGACATGACCGCCGAACAGCTGCACGCGCCT
             ************************************************************

ESTAII-TM    GCGGCCGGCGATCCACTGCTGCATCCGTCCTGGCTAGACAGCGCTCGCGACGCCTACTGC
ESTAII       GCGGCCGGCGATCCACTGCTGCATCCGTCCTGGCTAGACAGCGCTCGCGACGCCTACTGC
             ************************************************************

ESTAII-TM    CCGGCCGGGCTGGACCGCGCCGACCCGATGGTGTCGCCGCTGTTCGGCCAGCTCAAGGGC
ESTAII       CCGGCCGGGCTGGACCGCGCCGACCCGATGGTGTCGCCGCTGTTCGGCCAGCTCAAGGGC
             ************************************************************
```

(b)

```
EstATII-TM      1 MSRYVDELYRNPGQPGLRALLRGMLKLLFRGLIRPPVPFAVQALVLRLLTLGMPLARGVT
EstATII         1 MSRYVDELYRNPGQPGLRALLRGMLKLLFRGLIRPPVPFAVQALVLRLLTLGMPLARGVT
consensus       1 ************************************************************

EstATII-TM     61 RSTEQIAGRPCMWHRPAAGGNGRVLLYLHGGAFVIGSPQTHRGICSALASRGQFDVCALD
EstATII        61 RSTEQIAGRPCMWHRPAAGGNGRVLLYLHGGAFVIGSPQTHRGICSALASRGQFDVCALD
consensus      61 ************************************************************

EstATII-TM    121 YRLAPVHPAPAACDDAVAAYQALLQRGYAPAQITLIGDSAGGNLVLVTAQKLAALKLPLP
EstATII       121 YRLAPAHPAPAACDDAVAAYQALLQRGYAPAQITLIGDSAGGNLVLVTAQKLAALKLPLP
consensus     121 ***** ******************************************************

EstATII-TM    181 ASLVCFSPVTDMTAEQLHAPAAGDPLLHPSWLDSARDAYCPAGLDRADPMVSPLFGQLKG
EstATII       181 ASLVCFSPVTDMTAEQLHAPAAGDPLLHPSWLDSARDAYCPAGLDRADPMVSPLFGQLKG
consensus     181 ************************************************************

EstATII-TM    241 LPPLLLQVGEDEVLLNDSLRLAEAARAADVAVRLERYEDLWHVFQAHAGLLHSADAALQR
EstATII       241 LPPLLLQVGEDEVLLNDSLRLAEAARAADVAVRLERYEDLWHVFQAHAGLLHSADAALQR
consensus     241 ************************************************************

EstATII-TM    301 VVDFVNSAQTDSVS
EstATII       301 VVDFVNSAQTDSVS
consensus     301 **************
```

Figure S1. Est-ATII and Est-ATII-TM DNA and protein alignments. (a) . Est-ATII and Est-ATII-TM nucleotide alignment. The alignment shows the nucleotide substitution at position 377. (b) Protein alignment of Est-ATII and Est-ATII-TM showing the amino acid substitution at position 126.

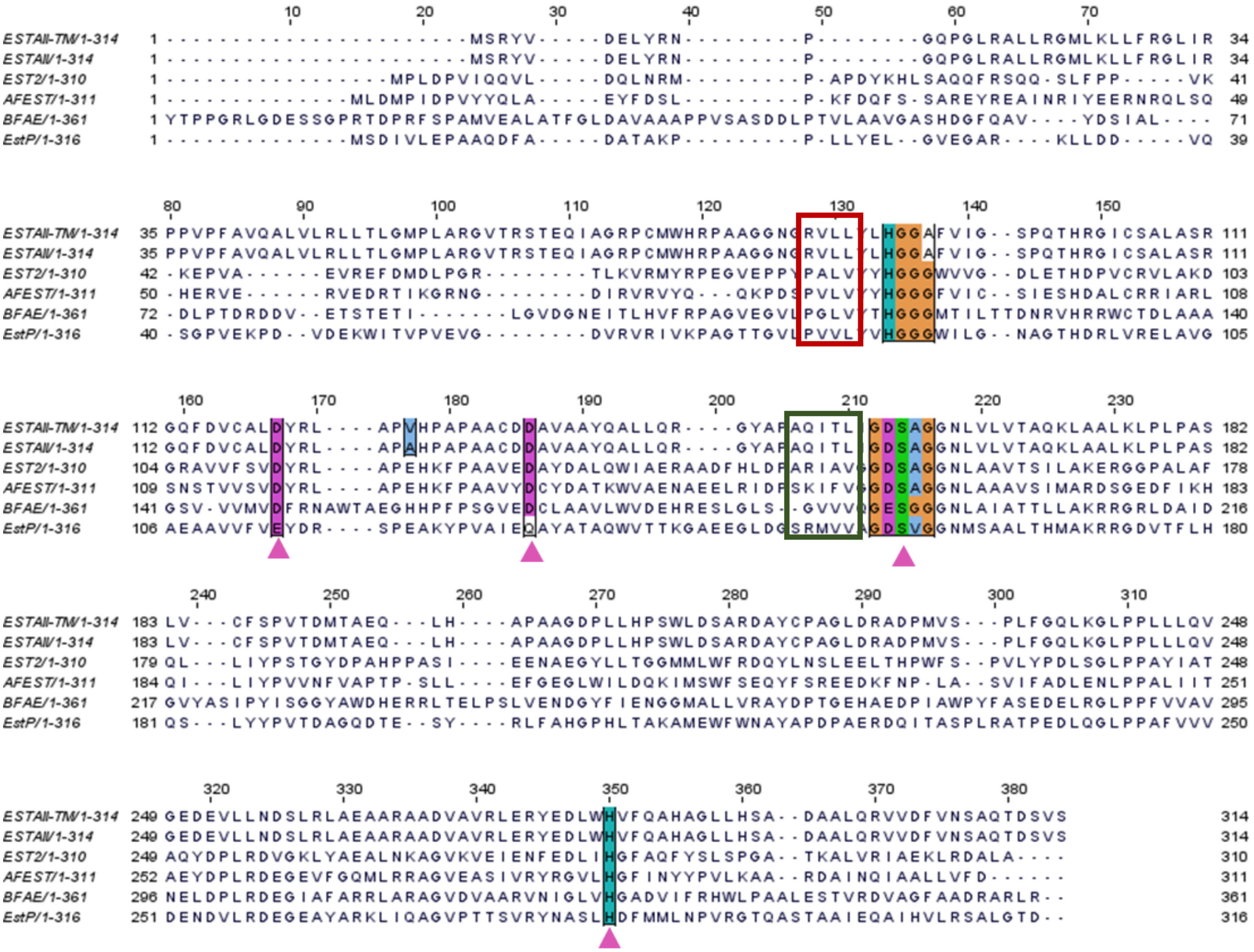

Figure S2 . Amino acids sequence alignments of EstATII, EstATII-TM, and enzymes from the HSL family. The typical HSL motif is indicated using a red rectangle. The Catalytic domain is boxed in the green rectangle, and the catalytic triad (Asp, His, and Ser) is emphasized with a pink triangle. The alignment was performed by Clustal Omega software, and the final image was generated by jalview. The accession numbers of the aligned sequences are for the following organisms: 1EVQ_A, carboxylesterase Est2 from *Alicyclobacillus acidocaldarius*, brefeldin A esterase from *Bacillus subtilis*; CAA37862, 1JJI (AFAST) Hyper-thermophilic Carboxylesterase from the *Archaeon Archaeoglobus fulgidus*, carboxylesterase (Est2) from *Alicyclobacillus acidocaldarius*, and EstP from *Pseudomonas putida.*

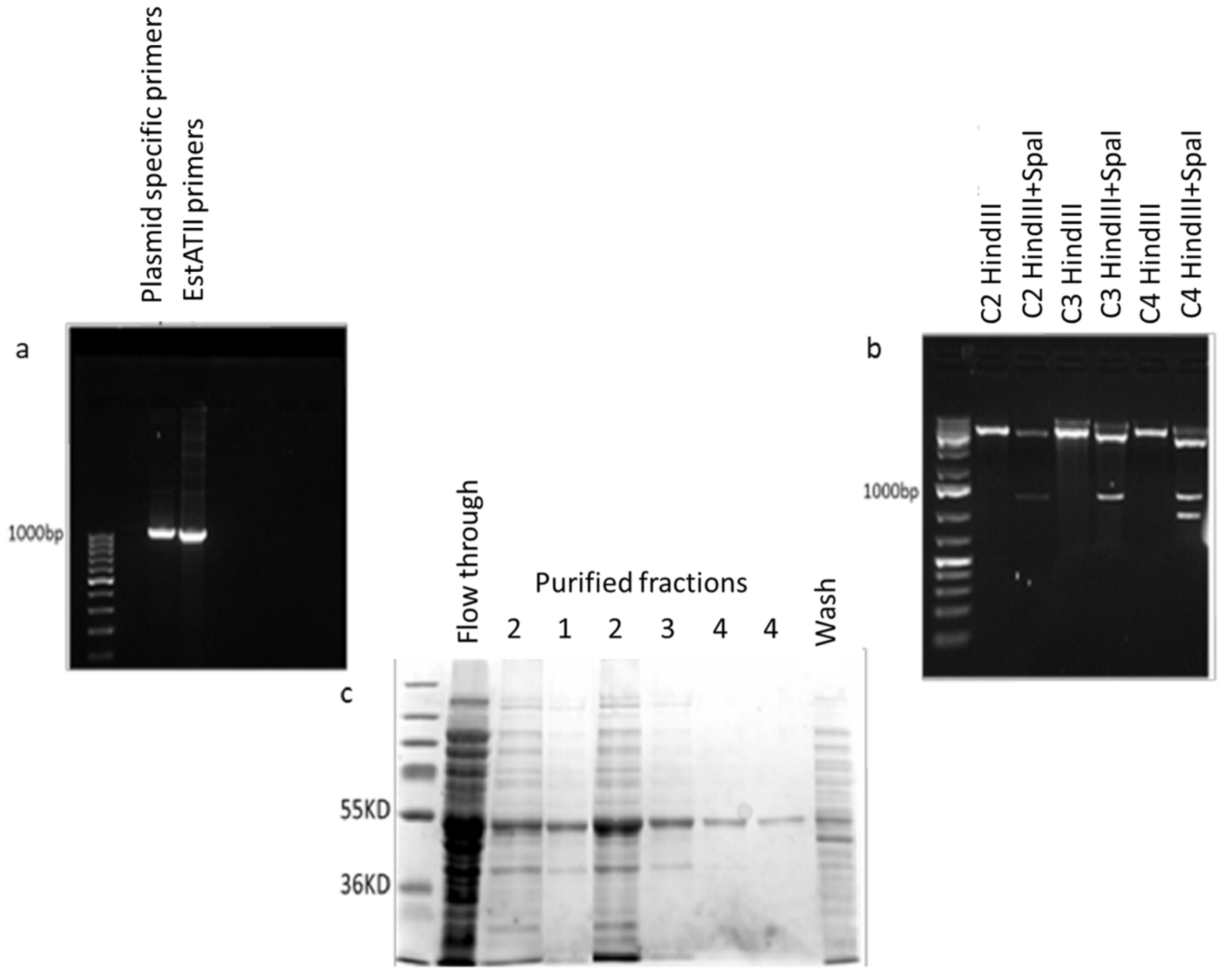

Figure S3. EstATII-TM cloning and expression. a) Agarose gel of the EstATII-TM PCR products using EstATII primer and T7 Primer sets. b) Agarose gel containing three recombinant plasmids digested with HindIII (single digestion) and HindIII /SpaI (double digestion). C2 and C3 are showing two clones that were cloned in the wrong orinatation, while C4 plasmid showed EstATII-TM in the correct orientation. c) SDS-PAGE of EstATII-TM: Lane 1: pre-stained protein marker, Lane 2: the flow-through after adding the cell lysate to the chromatography Nickel His-tag column, Lane 3 to Lane 8: the purified protein fractions (1,2,3 and 4), Lane 9: flow-through after washing the protein. EstATII-TM concentration: fraction/lane 1 = 0.229mg\ml, fraction/lane 3 = 0.220 mg\ml, and fraction/la 4 = 0.096mg\ml.

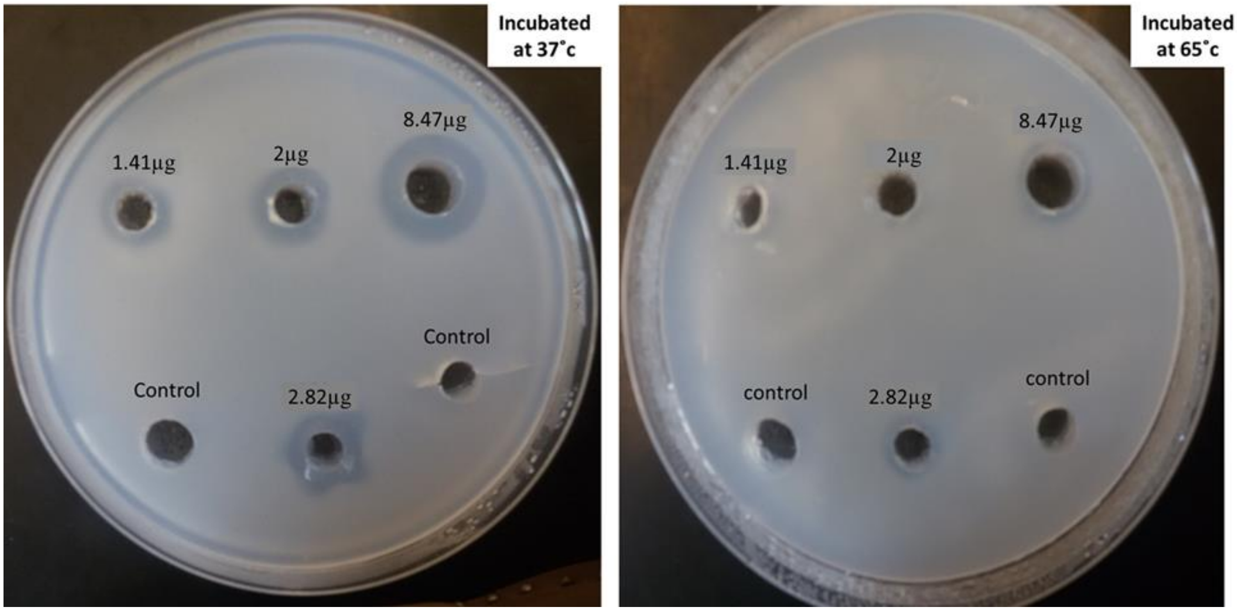

Figure S4. The qualitative assay of EstATII-TM using the cup-plate method and tributyrin (triglyceride) as a substrate. The control well in each plate contains sodium phosphate buffer without the enzyme. Each well contained the indicated concentration of the enzyme.

Table S1: Metal ions binding sites based on the Metal Ion-Binding site prediction and docking server (MIB) using the PDB ID 4XVC* for metal ion prediction.

Metal ion	Binding site in the enzyme	Binding potential
Cu^{2+}	30	1.8-2.8
Cd^{2+}	20	2-8
Mg^{2+}	13	1.9-4.1
Zn^{2+}	24	2.2-4.6
Fe^{3+}	30	1.6-2.7
Mn^{2+}	17	2.1-4
Hg^{2+}	17	2.3-5.9

* PDB ID 4XVC had the closest 3D structure to the predicted 3D structure of both EstATII-TM and EstATII when this analysis was made.
